# Residual beta cell function at diagnosis of type 1 diabetes in children and adolescents varies with gender and season

**DOI:** 10.1002/dmrr.2365

**Published:** 2013-01-08

**Authors:** U Samuelsson, B Lindblad, A Carlsson, G Forsander, S Ivarsson, I Kockum, Å Lernmark, C Marcus, J Ludvigsson, Better Diabetes Diagnosis (BDD) study group

**Affiliations:** 1 Department of Clinical and Experimental Medicine, Division of Pediatrics and Diabetes, Research Center, Linköping University HospitalLinköping, Sweden; 2 Department of Pediatrics, Queen Silvia Children's HospitalGöteborg, Sweden; 3 Department of Pediatrics, University Hospital MASMalmö, Sweden; 4 Department of Pediatrics, Lund University HospitalLund, Sweden; 5 Department of Molecular Medicine, Karolinska InstituteStockholm, Sweden; 6 Department of Clinical Sciences, Lund UniversityMalmö, Sweden; 7 Department of Pediatrics, Karolinska University HospitalHuddinge, Sweden

**Keywords:** C-peptide, children, type 1 diabetes, seasonal variation, gender, immune intervention

## Abstract

**Background:**

There are seasonal variations and gender differences in incidence of type 1 diabetes (T1D), metabolic control and responses to immune interventions at onset of the disease.

We hypothesized that there are seasonal and gender differences in residual insulin secretion already at diagnosis of T1D.

**Methods:**

In 2005, a national study, the Better Diabetes Diagnosis, was started to classify all newly diagnosed children and adolescents with diabetes. About 95% (3824/4017) of the patients were classified as T1D, and our analyses are based on the patients with T1D.

**Results:**

C-peptide was lower in younger children, 0–10 years of age (0.23 ± 0.20 nmol/L) than in older children, 11–18 years of age (0.34 ± 0.28 nmol/L) (*p* < 0.000 ). There was a seasonal variation in non-fasting serum C-peptide, significantly correlated to the seasonal variation of diagnosis (*p* < 0.01). Most children were diagnosed in January, February and March as well as in October when C-peptide was highest, whereas fewer patients were diagnosed in April and May when serum C-peptide was significantly lower (*p* < 0.01). The seasonal variation of C-peptide was more pronounced in boys than in girls (*p* < 0.000 and *p* < 0.01, respectively). Girls had higher C-peptide than boys (*p* < 0.05), especially in early puberty.

**Conclusions:**

Both seasonal and gender differences in residual beta cell function exist already at diagnosis of T1D. These observations have consequences for treatment and for randomizing patients in immune intervention clinical trials. Copyright © 2012 John Wiley & Sons, Ltd.

## Introduction

Immune intervention with glutamic acid decarboxylase (GAD) treatment of children and adolescents with recent onset of type 1 diabetes (T1D) seemed to delay the loss of endogenous C-peptide, at least in a phase II trial, and in some prespecified subgroups of a phase III trial 1,2. In both phases II and III studies, there was a significant efficacy in patients treated during early spring 2, which raises the question whether there is a seasonal variation disease process and/or beta cell function. Seasonal variation in the incidence of T1D is well known 3, although there is no clear explanation of this phenomenon. It has been speculated that the seasonal variation is related to virus infections 4. Other possible explanations include hormonal variation, variation in physical activity with more physical activity and better insulin sensitivity during summer and therefore less insulin requirement, variation in sun exposure and vitamin D or a simple explanation could just be less active observation of symptoms such as thirst and polyuria during hot summer months.

In the European Phase III GAD trial, the efficacy was also different in men and women with a significant efficacy in men but not in women 2.

Regarding gender differences, we and others have earlier noticed that there were significant differences in the degree of metabolic control between girls and boys with T1D 5. Thus, it is common that girls have higher HbA_1c_ in the ages of 10–18 years, which often has been taken as a sign of less good care of teenage girls. There are differences in age of puberty between girls and boys, differences in physical activity with more physically active boys 6, girls have more subcutaneous fat and have also an increased tendency of overweight during adolescence 7 parallel to an increased risk of both bulimia and anorexia 8. All factors mentioned previously may contribute to the less good metabolic control of girls with T1D, but are there differences also in disease process? Thus, we know that T1D is twice as common in boys as in girls after the age of 15 years 9, in contrast to other autoimmune diseases that tend to be more common in women than in men.

As both seasonal variation and gender seem to be involved in the course of T1D, we hypothesized that the disease process may be influenced leading to difference in residual insulin secretion already at diagnosis of T1D. Therefore, we decided to study these questions using data from almost 4000 newly diagnosed patients with T1D in a nationwide study in Sweden.

## Materials and methods

In 2005, a prospective national study, the Better Diabetes Diagnosis, was started in Sweden to classify all newly diagnosed children and adolescents with diabetes. Children below the age of 18 years with new onset diabetes are referred to a paediatric clinic. This cross-sectional prospective study is based on patients from all 43 Swedish paediatric clinics.

Diagnosis of diabetes is based on the American Diabetes Association criteria for diagnosis and classification of diabetes (i.e. casual plasma glucose > 11.1 mmol/L or a fasting plasma glucose > 7.0 mmol/L and symptoms of polyuria, polydipsia and weight loss) 10. In total, 4017 patients were included in this study. Questions on family history regarding diabetes and autoimmune disorders among first-degree relatives, symptoms and signs as well as height and weight were registered in Swedish National Paediatric Diabetes Registry, a national incidence and quality control registry 11. The diagnosis and classification of diabetes were initially based on clinical symptoms and signs, later on strengthened by information on diabetes-related autoantibodies, human leukocyte antigen-types, C-peptide, and in some cases maturity onset diabetes of the young genetics 12. About 95% (3824/4017) of the patients were classified as T1D (Table 1), and our analysis of difference in C-peptide between women and men is based on the 3824 patients with T1D.

**Table 1:** Type of diabetes in a Swedish nationwide population of children and adolescents

Type of diabetes	Number	Percent
Type 1	3824	95
Type 2	86	2
Maturity onset diabetes of the young	40	1
Secondary diabetes	30	1
Unknown type	13	0.4
Antibody negative	17	0.5
Another type	7	0.1
	4017	100

The Karolinska Institute Research Ethics Board approved the study, and informed consent from the parents was obtained.

### Determination of C-peptide

Serum C-peptide from the random non-fasting blood sample, taken at diagnosis before the first insulin injection, was measured at Linkoping University, Sweden, with a time-resolved fluoroimmunoassay (AutoDELFIA™ C-peptide kit, Wallac, Turku, Finland), with a detection level of 0.03 nmol/L. The sample was taken before the first insulin injection, mostly day 1. Each assay was validated by inclusion of a C-peptide control module containing a high, a medium and a low-level control (Immulite, DPC, UK). A 1224 MultiCalc® programme (Wallac) was used to calculate the levels of C-peptide.

### Statistical analysis

spss 18® (SPSS Inc., Chicago, IL, USA) was used for the analyses. Unpaired two-tailed Student's *t*-test and one-way analysis of variance was used. When there were indications of skewed distribution, Mann–Whitney *U*-test or Kruskall–Wallis test was used. Comparisons of groups were performed by crosstabs and chi-square (*X*^2^), or Fisher's exact test was used for proportions. To study whether there was a seasonal variation of diagnosis and/or a seasonal variation of C-peptide at diagnosis, an ordinary chi-square test [(observed (O) − expected (E))^2^/E] was used with 11 degrees of freedom. The mean C-peptide value each month was the observed value, and the mean value for all months was the expected value. Regarding the variation of diagnosis, the actual number of children was the observed value and the total number of children/12 was the expected value. *p* < 0.05 was regarded as statistically significant. The results are expressed as mean ± standard deviation.

## Results

**Figure 1:**
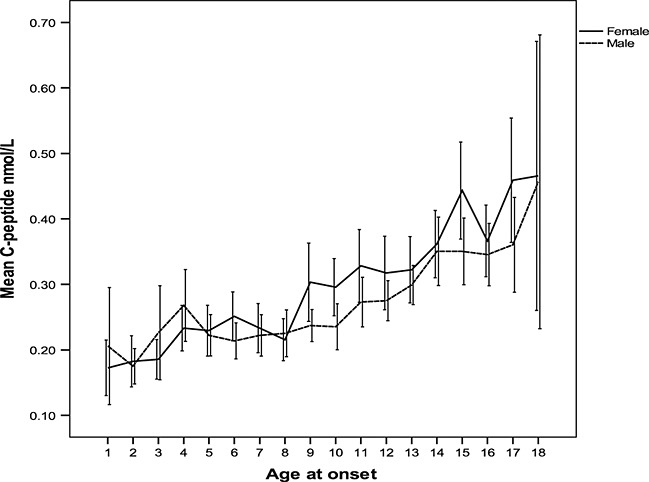
C-peptide in relation to age diagnosis of type 1 diabetes in girls and boys

**Figure 2:**
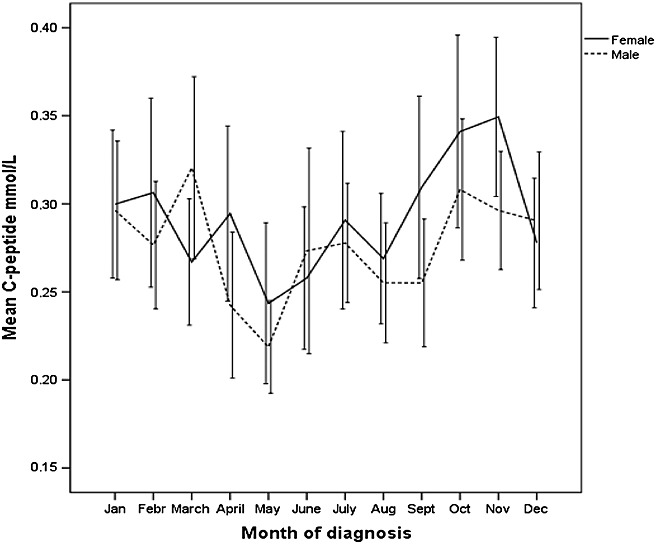
Seasonal variation of non-fasting serum C-peptide in children and adolescents with type 1 diabetes

**Figure 3:**
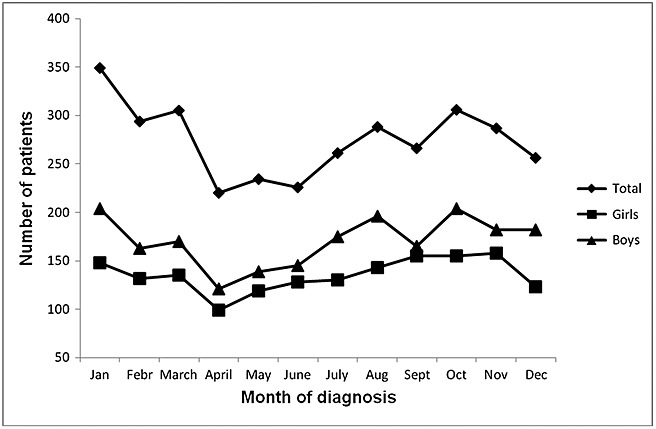
Seasonal variation of diagnosis of type 1 diabetes in boys and girls

C-peptide was lower in younger children, 0–10 years of age (0.23 ± 0.20 nmol/L) than in older children, 11–18 years of age (0.34 ± 0.28 nmol/L) (*p* < 0.000) (Figure 1). There was a clear seasonal variation over the year in non-fasting serum C-peptide at diagnosis (*X*^2^ = 25.4, *p* < 0.01) (Figure 2). This seasonal variation of C-peptide was significantly correlated to the seasonal variation of diagnosis (Figure 3) (*p* < 0.05, Spearman's two-tailed). Most children were diagnosed in January, February and March as well as in October when C-peptide was highest, whereas few patients were diagnosed in April and May when serum C-peptide also was significantly lower (*p* < 0.01). The seasonal variation was somewhat more pronounced for boys than for girls (*X*^2^ = 49.9, *p* < 0.000 and *X*^2^ = 27.3, *p* < 0.01, respectively, in total *X*^2^ = 71.2, *p* < 0.000) (Figure 3).

Girls had slightly higher C-peptide than boys (*p* < 0.05) (Table 2). The lower C-peptide in boys was associated with differences between the two sexes in symptoms and signs at diagnosis (Table 2).These gender differences were similar in all age groups (Table 3), but the difference in C-peptide was especially pronounced from 9 years of age (Figure 1). The gender differences did not explain the seasonal variation in C-peptide as both genders had about the same seasonal variation of C-peptide at diagnosis (Figure 2).

**Table 2:** C-peptide and some clinical differences between boys and girls at diagnosis

	Boys			Girls		
		
	*n*	Mean	SD	*n*	Mean	SD
HbA_1c_ (mmol/mol)	1913	92.2*	24.4	1528	96.2*	26.7
C-peptide (nmol/L, onset)	1838	0.28**	0.25	1426	0.30**	0.25
BMI-SDS	1838	−0.39	1.56	1448	−0.48	1.39
Age (year)	2020	10.2*	4.5	1588	9.4*	4.0
pH	1923	7.34	0.09	1511	7.34	0.10
BE (mmol/L)	1883	−4.1*	6.8	1492	−5.1*	7.5
*p*-glucose (mmol/L)	1950	27.3	9.1	1547	26.3	8.7
	*n*	Yes (%)			Yes (%)	
Weight loss	2131	1453 (68.2)		1693	1189 (70.2)	
Polydipsia	2131	1870 (87.8)		1693	1482 (87.5)	
Polyuria	2131	1897 (89)		1693	1482 (87.5)	
Other autoimmune disease	2131	80 (3.8)*		1693	110 (6.5)*	

BMI-SDS, body mass index standard deviation score; BE, base excess.

** *p* < 0.05.

* *p* < 0.01.

**Table 3:** C-peptide and some clinical differences between boys and girls at diagnosis in different age groups

		0–5 years		6–10 years		11–15 years		16–18 years	
					
	Sex	*n*	Mean ± SD	*n*	Mean ± SD	*n*	Mean ± SD	*n*	Mean ± SD
HbA_1c_ (mmol/mol)	Boy	385	77.9 ± 18.5	551	87.9* ± 21.2	758	99.7* ± 24.8	221	101.8 ± 25.4
	Girl	318	80.1 ± 19.1	554	95.4* ± 24.4	523	104.8* ± 27.3	135	103.6 ± 30.6
C-peptide (nmol/L, onset)	Boy	364	0.23 ± 0.24	538	0.23* ± 0.16	721	0.31** ± 0.26	216	0.36 ± 0.32
	Girl	292	0.21 ± 0.15	513	0.26* ± 0.23	495	0.35** ± 0.29	128	0.42 ± 0.29
BMI-SDS	Boy	359	−0.66 ± 1.37	528	−0.20 ± 1.6	728	−0.32** ± 1.5	224	−0.72 ± 1.7
	Girl	295	−0.56 ± 1.24	517	−0.36 ± 1.4	510	−0.52** ± 1.4	129	−0.54 ± 1.5
pH	Boy	383	7.36 ± 0.09	559	7.36 ± 0.08	746	7.33 ± 0.09	228	7.33 ± 0.09
	Girl	318	7.35 ± 0.09	538	7.35 ± 0.09	522	7.32 ± 0.11	130	7.34 ± 0.10
BE	Boy	377	−4.3** ± 6.6	548	−3.3* ± 6.0	739	−4.5* ± 7.4	221	−4.1 ± 7.3
	Girl	317	−5.3** ± 6.8	534	−4.5* ± 7.2	515	−5.8* ± 8.2	129	−4.1 ± 7.8
*p*-glucose (mmol/L)	Boy	389	26.6 ± 7.8	560	26.9 ± 8.4	763	27.7 ± 9.6	231	28.5* ± 10.5
	Girl	324	27.0 ± 8.3	555	26.1 ± 8.0	529	26.6 ± 9.6	134	24.9* ± 8.9
		*n*	Yes (%)	*n*	Yes (%)	*n*	Yes (%)	*n*	Yes (%)
Weight loss	Boy	411	52.3**	580	69.1**	790	80)	239	83.7
	Girl	338	61.2**	569	75.4**	541	81.5	140	77.9
Polydipsia	Boy	411	90.3	580	92.4	790	93.2	239	92.5
	Girl	338	92.9	569	93.3	541	93.3	140	92.1
Polyuria	Boy	411	92.2	580	94.7	790	93.7	239	93.3
	Girl	338	93.2	569	93.3	541	93.2	140	92.1
Other autoimmune disease	Boy	411	3.6	580	5.6**	790	5.6 *	239	2.5**
	Girl	338	5.9	569	9.1**	541	9.1 *	140	7.9**

BMI-SDS, body mass index standard deviation score; BE, base excess.

** *p* < 0.05.

* *p* < 0.01.

C-peptide at diagnosis was related to symptoms and signs at diagnosis (Table 4). Thus, children without polyuria and polydipsia at diagnosis and children without weight loss at diagnosis had higher C-peptide values than children with such symptoms and signs.

**Table 4:** Some symptoms and signs at diagnosis, as well as family history of diabetes in relation to C-peptide at diagnosis

		C-peptide, diagnosis			
				
Symptoms/history		Number	Mean	SD	*p*-value
Polyuria	Yes	3067	0.27	0.21	<0.000
	No	139	0.71	0.51	
Polydipsia	Yes	3043	0.26	0.21	<0.000
	No	154	0.70	0.51	
Weight loss	Yes	2397	0.25	0.19	<0.000
	No	701	0.42	0.37	
pH <7.3	Yes	513	0.18	0.15	<0.000
	No	2606	0.30	0.24	
BMI-SDS ≤0	Yes	1872	0.24	0.19	<0.000
	No	1125	0.37	0.32	
Other auto	Yes	173	0.32	0.31	n.s
immune disease	No	3268	0.29	0.26	
T1D in family	Yes	448	0.37	0.28	<0.000
	No	2993	0.27	0.26	
T1D in grandparents	Yes	220	0.34	0.25	0.01
	No	3221	0.29	0.27	
T2D in relatives	Yes	1121	0.31	0.27	0.01
	No	2320	0.28	0.27	

BMI-SDS, body mass index standard deviation score; T1D, type 1 diabetes; T2D, type 2 diabetes.

Children with T1D and who had T1D in the family, in grandparents or in first-degree relatives in general had a higher mean C-peptide at onset than T1D children without T1D in the family or among relatives (Table 4). T2D in the family, in grandparents or in first-degree relatives had no such relation to C-peptide at diagnosis in the T1D patients. Furthermore, children with low pH (<7.3) and low body mass index standard deviation score at diagnosis had low mean C-peptide value. Co-morbidity with another autoimmune disease did not influence level of C-peptide (Table 4).

## Discussion

As shown already long time ago 13, younger patients had significantly lower C-peptide than patients diagnosed as teenagers. It is also reasonable that patients with T1D in the family are diagnosed a bit earlier with higher C-peptide. Type 2 diabetes in the family was not associated with higher C-peptide at diagnosis of T1D, nor an autoimmune disease beside T1D. As expected, there are also correlations between C-peptide, symptoms and signs at diagnosis.

The focus of this study was to elucidate if seasonal and gender differences of beta cell function might be part of the explanation why immune intervention studies have shown different results related to these parameters. Our results from this nationwide large unselected patient's population support the hypothesis that there, at least in Sweden, are seasonal variations in the disease process with differences in residual beta cell function already at diagnosis. Thus, we found not only a seasonal variation of diagnosis, which is well known 14, but also a clear seasonal variation of C-peptide at diagnosis with lower C-peptide in patients diagnosed in those months (April and May) when the incidence was lowest. We measured non-fasting C-peptide influenced by actual food intake, but there is no evidence that seasonal variation of food intake could explain our findings. Our results support that season may play a role for the precipitation of manifest T1D and/or course of T1D and may therefore also play a role for the effect of immune interventions. Why children diagnosed during April and May are fewer, but have significantly lower C-peptide, is unclear, but could, for instance, be related to infections 15,16. There is also a known seasonal variation in the immune system among children 17, which might to some extent be related to seasonal variation of vitamin D, associated with exposure to sun and light. The seasonal variation both in immune system and in residual beta cell function may be part of the explanation why the effect of GAD treatment in newly diagnosed T1D has shown best effect in patients diagnosed during early spring 1,2.

In light of the known gender differences in both incidence of T1D and metabolic control, the fact that immune intervention has shown different effects in women and men (2) is perhaps reasonable. It has earlier been shown that GAD antibodies response and its related C-peptide decline is more pronounced in women than in men 18. Our results show that there is a difference between girls and boys already at diagnosis of diabetes. Difference in immune function between girls and boys 19–21 may partly explain why girls have significantly higher C-peptide at diagnosis. The difference exists in all ages but becomes especially pronounced in the ages 9–11 years when girls go into puberty, somewhat earlier than boys, and therefore with increasing insulin resistance may get their manifest diabetes at a higher C-peptide production.

In conclusion, seasonal variation and gender differences in both immune function and T1D are well known, and evidently differences in beta cell function exist already at diagnosis of T1D. Even though it may be too early to tailor-cut individual treatments for patients with T1D, we have to be aware of whom we treat and in what situation when trying to improve the effect of immune interventions in T1D.
